# “I Do Not Believe We Should Disclose Everything to an Older Patient”: Challenges and Ethical Concerns in Clinical Decision-Making in Old-Age Care in Ethiopia

**DOI:** 10.1007/s10728-024-00494-y

**Published:** 2024-10-01

**Authors:** Kirubel Manyazewal Mussie, Mirgissa Kaba, Jenny Setchell, Bernice Simone Elger

**Affiliations:** 1 Institute for Biomedical Ethics, University of Basel, Bernoullistrasse 28, 4056 Basel, Switzerland; 2 School of Public Health, Addis Ababa University, Addis Ababa, Ethiopia; 3 School of Health and Rehabilitation Sciences, The University of Queensland, Brisbane, Australia; 4 Center for Legal Medicine, University of Geneva, Geneva, Switzerland

**Keywords:** Decision-making, Older adults, Religion, Lying, Family involvement, Ethical issues

## Abstract

Clinical decision-making in old-age care is a complex and ethically sensitive process. Despite its importance, research addressing the challenges of clinical decision-making in old-age care within this cultural context is limited. This study aimed to explore the challenges and ethical concerns in clinical decision-making in old-age care in Ethiopia. This qualitative study employed an inductive approach with data collected via semi-structured interviews with 20 older patients and 26 health professionals recruited from healthcare facilities in Ethiopia. Data were analysed using reflexive thematic analysis. Our analysis identified three key themes. First, participants highlighted perceptions that older patients’ religious beliefs interfere with the clinical decisions both older patients and health professionals make. Second, older patients often receive limited information from health professionals about their diagnosis and treatment. Third, families of older patients appear to strongly influence clinical decisions made by older patients or health professionals. This research enhances the understanding of clinical decision-making in old-age care within Ethiopia, a context where such research is scarce. As a result, this study contributes towards advancing the deliberation of ethical dilemmas that health professionals who work with older patients in Ethiopia might face. A key implication of the study is that there is a need for more ethics and cultural competence training for health professionals working with older patients in Ethiopia.

**Supplementary Information:**

The online version contains supplementary material available at 10.1007/s10728-024-00494-y.

## Background

Decision-making is a crucial concept in clinical practice. While the broader system of decision-making in healthcare can involve policymakers and programme managers [1], in daily clinical practice, decision-making mainly involves health professionals, patients and family caregivers in any patient context [2]. Additionally, the quality of decision-making significantly determines the quality of care, patient safety and treatment outcomes [3]. Health professionals have the responsibility to make carefully considered decisions by, for example, respecting patients’ autonomy and involving them in the decision-making process. Conversely, patients should actively participate by providing relevant medical information and evaluating treatment options to ensure that decisions positively influence treatment outcomes. Poor decision-making, regardless of whether it happens at the level of health professionals or patients, could lead to adverse health outcomes such as avoidable complications and increased mortality [4, 5]. Factors that may affect the quality of clinical decision-making include patients’ beliefs, the cultural context of clinical practice, health professionals’ competence, and decision fatigue [3, 5, 6]. These factors, coupled with resource and expertise limitations in various healthcare contexts [7–11], could further complicate the implementation of conventional ethical principles. A comparative study between China and the US, for example, elaborates on how cultural nuances significantly influence the clinical decision-making process [12]. Similarly, various studies in non-Western contexts demonstrate that communal decision-making often takes precedence over individual autonomy [13–15].

Clinical decisions concerning older patients are often complex [16] and present unique challenges for all involved—such as health professionals, older patients and informal caregivers—due to factors such as medical complexities, uncertainties about prognosis and treatment, communication challenges, and the involvement of multiple family and other stakeholders [17, 18]. Cultural and religious beliefs further complicate these decisions, as older adults usually hold such beliefs more strongly than younger people [19, 20]. These and other barriers make clinical decision-making with older patients particularly difficult. Studies show that poor clinical decision-making can be more common when working with older patients [16, 21] and can result in serious consequences such as severe illness and mortality [5]. Such findings highlight that clinical decision-making with older patients could involve serious ethical concerns such as harm and breach of autonomy, which warrant careful consideration. This challenge could be more complicated in African contexts where, for example, involvement of extended families in decision-making processes, combined with traditional healing practices, further complicate the ethical landscape and warrant the need for culturally adapted decision-making frameworks [22, 23].

In Ethiopia, clinical decision-making is likely to be a particularly complex and challenging process. A recent study on decision-making at each level of the Ethiopian health system reports that there is little routine use of evidence-based information for clinical decision-making in Ethiopia, which might negatively affect accountability and the quality of decision-making [24]. In addition, cultural norms and religious practices including belief in supernatural powers, fasting and the use of holy water as a means of cure frequently conflict with “modern” or Western medicine [25–27] and pose an additional challenge to clinical decision-making processes. Similar challenges have been observed in other African countries where traditional medicine and religious beliefs significantly influence patient care and clinical decision-making [22]. Furthermore, there is a lack of culturally specific ethical guidelines and medical ethics training, leaving health professionals with little guidance. As a result, they must navigate these challenges and confront ethical dilemmas while attempting to make the ‘right’ clinical decisions [28–31], often without access to local decision-making frameworks.

When it comes to old-age care in Ethiopia, clinical decision-making is a complex process for both older patients (and their informal caregivers) and health professionals, and this complexity warrants deeper investigation. This complexity arises, in part, due to the health system’s limited preparedness for old-age care despite the country’s older population is rapidly increasing. For example, according to the UN 2015 World Population Report, the country’s older population accounted for 5.2 million (approx. 5% of the total population) in 2015 [32]. Similarly, the Ethiopian Central Statistical Agency predicts that the older population will make up 7.3% of the total population by 2037 [33]. There have been a few notable policy efforts from the Ethiopian government to ensure the health and general well-being of older adults—for example, ratifying the AU Protocol on the Rights of Older People in July 2020 [34] and developing the 10 year National Plan of Action on Older Persons in June 2006 [35]. However, many older adults in the country continue to experience health and wider socio-economic challenges [36–40]. There remain many research and policy gaps in deliberating and addressing challenges in providing healthcare for older adults in the country. More specifically, research investigating the challenges and ethical implications of clinical decision-making in old-age care is scarce. Available studies either focus on decisions regarding co-residential family care among rural older adults [41], shared decision-making among adults in general [26], or autonomous decision-making in the context of women and maternal health [42–44]. Thus, based on interviews with both older patients and health professionals, the aim of this study was to explore challenges and ethical concerns in clinical decision-making in old-age care in Ethiopia.

## Methods

### Design

An inductive exploratory approach was used as there is limited knowledge of the study context and topic [45, 46]. The study obtained ethical approval from the Ethics Committee of the University of Basel in Switzerland and the City Government of Addis Ababa Health Bureau in Ethiopia. All participants provided informed consent and their data was anonymised during the analysis.

### Participants

Participants were 20 older patients and 26 health professionals recruited in person by the first author (KMM) from and through health facilities using purposive sampling techniques. This sampling technique is useful for choosing participants based on relevant knowledge or experience [47, 48]. In this study, old age was defined as 60 years and over, in accordance with the UN definition for sub-Saharan countries [32] and Ethiopia's formal retirement age of 60 [35]. In addition to these age-based criteria, older patients in this study had experience receiving medical treatment in Ethiopia, and were able to communicate verbally and give written informed consent [49]. Health professional participants were working in a healthcare facility during the interview period and had professional experience caring for older adult patients (as defined above) in Ethiopia. Health professionals were selected from four government and two private healthcare facilities in Ethiopia’s capital, Addis Ababa.

All interviewees received hard copies of informed consent forms and all relevant information prior to their participation. Participants were briefed on the researcher's professional background, encompassing their credentials and expertise in qualitative research methodologies. However, personal motivations or objectives behind the research were withheld to mitigate any potential bias on participants' responses. Furthermore, the interviewer (KMM) verbally explained to participants the study's purpose and the content of the informed consent, including their right to withdraw their participation and/or data at any stage of the study without providing a reason, and asked them to reformulate their answers to ensure they fully understood what they were consenting to. In certain instances, especially with interested participants, this was undertaken a few days prior to the interviews. This approach not only facilitated the establishment of a professional rapport but also fostered a professional relationship with the participants. The participants then signed and returned the written informed consent, and the researcher conducted the interviews in person. Participation in this study was entirely voluntary, and participants received no remuneration because they incurred no costs as a result of their participation [50, 51]. The total number of participants in this study was 46 (26 health professionals and 20 older adults), which was determined after iterative analyses indicated that it was large enough to obtain a variety of perspectives and a rich understanding of participants' experiences and perceptions [52].

### Data Collection

We conducted semi-structured interviews at the participating healthcare facilities between March 3, 2021, and November 28, 2021. At the time of the interviews, the interviewer (KMM) was a male doctoral researcher with prior training and experience in qualitative interviewing techniques. All interviews took place within the chosen healthcare facilities, in secluded rooms where only the interviewer and participant were present. The interview guide was developed in English, the medium of communication among project members, and the interviews were conducted in Amharic, Ethiopia's official language and the interviewer's first language. Two pilot interviews were conducted, one with an older adult and one with a health professional, to ensure that the interview guides were understandable to participants and that the questions were open-ended enough to allow for discussions [53]. None of the information from these pilots was used in this study. Minor changes were made in response to pilot feedback, such as avoiding or elaborating on medical terms, locating more appropriate Amharic translations of ethical concepts, and broadening opening questions to facilitate probing and further discussions.

The interview guide questions aimed to capture the older adults' and health professionals' perspectives on: (1) how the religious beliefs of older patients influence clinical decisions, (2) the disclosure of clinical information to older patients concerning their diagnosis and treatment, and (3) family involvement in clinical decision-making (see Additional file 1). Interview averaged 1 h (range: 15–107 min). Except for two interviews, all were audio-recorded with participants' verbal consent. Because two participants declined to be voice recorded, the interviewer took written notes on their responses. KMM transcribed the recordings verbatim into English and anonymised them. MK and KMM reviewed the transcripts for quality and translation accuracy.

### Data Analysis

Data were entered into the MAXQDA-2022 software, which was used to store transcripts and facilitate the analysis process [54]. The transcripts were not provided to participants for review or amendment, primarily because of resource constraints and to mitigate any possible emotional strain or unease related to revisiting delicate subject matter. Nevertheless, to ensure credibility, the research team rigorously employed various techniques during data analysis, including extensive checks for transcription and translation accuracy. We used Braun and Clarke's reflexive thematic analysis approach, which consists of six phases: familiarisation with the data, generation of initial codes, theme search, theme review, theme definition and naming, and report production [55]. We used this approach after reading Braun and Clarke's recent critical reflection on the use of thematic analysis for greater rigour [52]. Recognising that "TA [thematic analysis] refers to a cluster of sometimes conflicting approaches," we specifically followed the reflexive TA approach, in which coding is open and themes are the final outcome of the analysis [52]. In light of this, and using the inductive content analysis approach [56], KMM began by open-coding the data with contributions from all co-authors. KMM read through the transcripts again, revised the codes, and classified them into tentative themes based on comments from the rest of the research team on the initial coding stage. The codes were then revised by two authors (JS and KMM), and KMM created the final coding tree. The reflexive approach [52] we employed also helped us to understand our perspectives would have influenced the data analysis and interpretations. However, we tried to be aware of our assumptions and are a mix of genders, nationalities (two Ethiopians, one Swiss, one Australian) and academic disciplines (bioethics, public health, physiotherapy, sociology). All authors were involved in commenting on the codes and the analysis process to provide a multifaceted and balanced view of the data. The analysis yielded two themes and five subthemes, on which all authors eventually agreed.

## Results

A total of 20 older adults and 26 health professionals were interviewed for this study. The older adults ranged in age from 60 to 87 years, with the majority being younger within the age group (only 3 were over 80 years). Among the health professional participants, nurses were the most common professionals (46.1%), followed by general practitioners (19.2%), internists (11.5%), public health officers (7.7%), and other professionals. Participants’ demographic characteristics are presented in Table 1. Our analysis of 46 interviews (20 older patients and 26 health professionals) resulted in three major themes: (1) religion interfering with older patients’ decisions to adhere to “modern” medicine, (2) limited informed consent, and (3) strong family involvement.

**Table 1:** Demographic information of participants

Older adults	
Age groups	Number of participants and percentage
60–64	7 (35%)
65–69	3 (15%)
70–74	2 (10%)
75–79	5 (25%)
80–84	1 (5%)
85–89	2 (10%)
Gender: 11F, 9 M	Total: 20

Health professionals	
Profession	Number of participants and percentage
Nurse	12 (46.1%)
Physicians and specialists	10 (38.5%)
Others (health officer, psychiatrist, etc.)	4 (15.4%)
Gender: 16F, 10 M	Total: 26

### Religion Interfering with Older Patients’ Decisions to Adhere to Modern Medicine

Several of our participants asserted that a sizable number of older patients hold strong religious beliefs concerning illness and cure and lack trust or interest in “modern” medicine [participants used the Amharic term “zemenawī ḥikimina” which can directly be translated as “modern medicine”]. This ranges from being against modern medicine as a whole to indifference towards specific aspects. One older patient gave the following general comment: *“There are those older people who hate modern medication and say ‘what is the use of medication for me after this age’? They may consider getting treated in hospitals as a sin.” (OP18)* Another older patient added: *“I do not want to go for follow-up. I leave my health condition as it is and wait for God. I will die like this if needed.” (OP1).*

Further elaborating on the beliefs some older patients have against modern medicine, a few health professionals gave examples of specific treatments that older patients refused to accept due to religious values. For example, one health professional referred to a specific religious denomination and said: *“In Orthodox Christianity, there is the prohibition of removing a body part. There were older patients who died because they refused amputation. They said ‘I want to die with my full body’”. (HP9)* Similarly, another health professional added: *“Most of the time the monks in the Orthodox church are challenging. When they get really old, they start losing hope and refuse medical treatments. They say ‘what am I doing after this age and why is God not taking me?’” (HP3)* Other health professionals further elaborated on how specific religious practices such as fasting could affect older patients’ decisions to adhere to prescribed treatments. Although this could be the case among younger patients as well, participants claimed it is a more common and serious issue among older patients due to their tendency, as some health professionals claimed, to adhere more strongly to religious practices. One health professional’s response summarises this: *“Older patients, both in Islam and Christianity, are very strict on fasting. There are medicines that must be taken in the morning and that do not go with fasting. This affects their treatments.” (HP3).*

Not all older patients expressed a lack of interest in modern medicine; a few were supportive. For example, one older patient said: *“When I get sick, I do not do anything else but go to the hospital. I do not like the traditional and other means of treatment.” (OP6)* Another added: *“I prefer hospitals. I even gave birth to all my children in hospitals.” (OP7)* There were no discernible patterns in these data to suggest that education or economic background of older patient participants influenced their attitudes toward modern medicine.

In addition to religious convictions, older patient participants expressed a few other reasons for not wanting ‘modern’ medical approaches. When making such statements, they always referred to religious practices as their preferred option. For one older patient, unsuccessful treatment experiences and treatment fatigue constitute one of the reasons: *“I love using holy water. It heals me from my illnesses and there is nothing else that can help. I tried many hospitals but without any change. I let God’s will to happen and die just as I am”. (OP1)* Another older patient gave a similar response and added an economic aspect: *“One doctor prescribed me injections and big tablets. They did not cure me but my condition worsened. I do not even have pocket money. So, it is better to go to church and pray to God.” (OP12)* Thus, treatment fatigue and financial problems were among the reasons some older patients decided against using modern medicine.

Some health professionals reported on how they respond to older patients who refuse treatment due to their religious beliefs. The underlying principle foregrounded by all of the health professional participants was respecting the wishes of the patient, as one health professional asserted: *“We do according to their interest. What is expected from me is explaining and the choice will be the patient’s.” (HP9)* However, further conversations with a few health professionals revealed that in contrast to this principle, were attempts to persuade older patients to adhere to treatments. For example, according to one health professional, one method is explaining that religion and modern medicine can go together: *“So one thing I do is explain that God is the source of knowledge in modern medicine and that they can still worship God while taking their medication. But explaining this is still difficult.” (HP21)* An additional technique another health professional added was being very polite and using words that can create emotional intimacy.

Our main challenge is those who fast. So instead of simply telling them to stop fasting, I say ‘mother, father, this medicine prevents suffering from you. I can kneel down and pray with you if that helps.’ So being polite helps to convince them. We have a saying that goes ‘the one below God [in terms of hierarchy/power to save people] is the doctor’, right? (HP5)

Lastly, as one health professional summarised it—*“I invite healthcare professionals from the same religion to come and explain to them” (HP1)*—an additional strategy we identified was inviting colleagues (other health professionals) from the same religious denomination to come and explain to the older patient.

### Limited Informed Consent

#### Limited Information Provided to Older Patients

Most of our participants also discussed how much information older patients receive concerning their diagnosis and treatment. Most of the older patients who commented on this criticised the lack of information from health professionals regarding their treatment. For example, one older patient said: *“If we refuse treatment, it is considered we are against modern medicine. Health professionals do not think that it might be because they have not explained to us* [about the treatments*]. Very few explain but most do not. They do not have time and competence.” (OP10)* Another added by stressing that older patients might not ask when things are not clear: *“They [health professionals] do not tell you your status, what will happen, *etc*. I am very conscious and ask. But other older patients fear doctors or say ‘I do not have knowledge’, so they do not ask.’” (OP14)* Similarly, one health professional expressed a criticism by referring to a case they encountered: *“I remember an older patient who received kidney surgery. His kidney was removed but he did not know. I understand the patient load and time pressure but still he deserves to know about his body. This was a breach of ethics for me.” (HP26).*

An additional concern from a few older patient participants was that information is not delivered in a way they can easily understand. One older patient said: *“the other problem is the doctors do not explain to us in a language that we understand.” (OP14)* This did not refer to using a language older patients do not speak but rather that the health professionals used medical terms unfamiliar to patients or delivered the information too rapidly. Another older patient participant further illustrated this by also highlighting the consequences: *“The way information is told to older patients is not based on their level of understanding. So they end up not making the correct decision and refuse surgery or taking medicines, *etc. *and suffer more as a result.” (OP15).*

In the first theme where we discussed the potential influence of religion on decision-making among older patients, health professionals at times said that they took additional time and used different techniques to explain the benefits of the suggested treatments and to increase patients’ adherence to treatments. The present theme indicates that health professionals often fail to provide older patients with adequate relevant information. Although these two findings might seem somewhat contradictory, they indicate that it is possible that while health professionals may show more commitment to giving more information to those older patients who refused treatment, they seemed to provide less information and spend less energy on those whom they think are adhering to treatment.

#### White Lies and Non-Disclosure

We asked the health professional participants what and how much information they believe should older patients receive concerning their diagnosis and treatment. Their responses frequently cited the age of older patients as the primary reason for withholding some information. One health professional summarised the issue: *“due to their age, the disclosure of medical information to older patients should be adjustable and thus, they should be informed in a way that does not affect their health progress” (HP19)*. Another health professional elaborated, sharing their experience with an older patient diagnosed with COVID-19.

There was an 80 year-old we diagnosed with COVID. We discussed this with the family and decided not to tell him. So we agreed to continue the treatment by combining herbal medicine like garlic, ginger, damakese [Ocimum Lamiifolium] with azithromycin and dexamethasone. He recovered and even drives a car now. […] It is very difficult to tell someone of his age that he has COVID, so I do not believe we should disclose everything to an older patient. (HP5)

This example suggests that the justification for lying to older patients was the perceived potential benefit to their health. One health professional further elaborated on this by comparing older and younger patients.

When you tell young patients about their illness, risks, etc honesty and letting them know everything must be a priority because they should do things that would benefit the treatment. But for older patients, I think you should not tell the truth if it does not add anything positive or if it hurts them. So, discussion with the family or caregivers should take priority. This could be wrong, but in the end, it should not be a problem as long as it benefits the patient. (HP19)

Other health professional participants provided more examples of the benefits of lying when working with older patients. For example, one health professional mentioned the case of older patients with HIV and stressed the potential of white lies in increasing treatment adherence: *“Older people are tough and may refuse to accept their HIV status. So, you tell them it is another disease and they start treatment. But you systematically work with the family. I had such older patients and the results were great.” (HP4)* Another health professional added: *“I do not know if it is ethical, but once we gave an older patient blood, covering it and telling him it was glucose” (HP3)*.

On the contrary, a few participants did not feel comfortable lying to older patients. One health professional said: *“Older patients are often protected from knowing about their own diagnosis. Last time we had an older woman who was literally going to breast surgery but no one told her about that. Perhaps it was to protect her, but I did not understand the reasoning.” (HP13)* Given that this health professional was the only non-Ethiopian participant (with a Western background), their different cultural views on family, autonomy, decision-making, and responsibilities likely contributed to their differing perspective. The next (final) theme regarding family involvement has some related insights.

### Strong Family Involvement

Several participants from both older patients and health professionals emphasised the role of the family in influencing older patients’ health-related decisions. The decision-making power of the family was discussed referring to older patients who had the mental capacity to make autonomous decisions. One health professional summarised the family’s power as follows: *“I think in Ethiopia, the family ultimately decides. Sometimes the older patients say they do not want treatment and want to die but the family disagrees and the patient gets treated. The family is stronger than the patient and also the health professional.” (HP13)* There were similar responses from most of the older patients who commented on this topic. For example, when asked about a moment when they visited a health facility without their interest, one older patient said: *“Even today, I came here [to the hospital] only because my daughter begged me. So, I came just to make her happy although I did not want to.” (OP7)* In the same vein, one health professional added a case they witnessed where the family overrode a decision an older patient made and how the patient dealt with the tension: *“There was one case where the family influenced an older woman patient to undergo amputation although she did not want it. She refused but finally, when the family insisted too much, she became confused, tired of resisting, and ultimately underwent the amputation.” (HP9)* However, perhaps due to these cultural expectations, no participants reported feeling forced to choose a treatment plan.

Our participants further explained why family usually has power over the decisions older patients make regarding their health care. The major reason was that the family—more specifically children—invest significantly in finances, time, and other resources to care for their older family members, which appears to give them the authority to influence the older family members' health decisions. As one older patient put it, *“when the older person needs medical attention, the entire family gets affected” (OP16)*. For example, some older patients said that their children usually take care of medical expenses and therefore, they hesitate to go against the suggestions their children make concerning treatment options. The response from one older patient summarises this: *“When I get sick, I call my younger son. He is the one who gives me money. So, he says, ‘Go to the hospital and take medicine. I will send you money for that.’ So, I accept his order [laughs].” (OP6)* Another older patient said that they would agree with their children’s suggestion to protect them emotionally: *“Sometimes I say I do not want to go to the hospital but my children continue to insist and finally take me. I agree to go not to hurt my children’s feelings.” (OP20)* A few health professionals also gave similar comments concerning the role of the family. One health professional said: *“the family can be stubborn out of fear of losing the older patient” (HP6)*. Another further elaborated on the family’s commitment and how that could give it authority over the decisions an older patient makes:

In Western countries, there are nursing homes for older people so the family may not be involved so much. But here in Ethiopia, I get amazed to see people quit or reduce their job and do everything to stay at home to care for their older family members. Culturally speaking, this is opposite from Europe or elsewhere and is very admirable, I think. […] But then sometimes you make the older patients suffer for the family. (HP13)

Finally, we captured some opinions from a few of the health professional participants concerning the implications strong family involvement has on their work with older patients. One health professional said: *“you treat the family more than you treat the older patient” (HP12)*. Another added: *“what makes working with an older patient difficult is also family members” (HP1)*. An additional health professional participant further explained how they reacted to this challenge, recalling an argument with a family member: *“I shouted at one family member saying ‘get out of my office and go to another doctor if you want’ [laughs]. He wanted me to decide something about an old lady. I do not remember the case.” (HP8)* While family involvement and sacrifice were viewed as admirable and noteworthy, a few health professionals argued that it posed challenges to the decision-making process.

## Discussion

This inductive qualitative study, based on interviews with older patients and health professionals in Ethiopia, explored the challenges and ethical concerns in clinical decision-making in old-age care. In a context like Ethiopia, where research on clinical decision-making for vulnerable population groups is limited, the findings contribute towards advancing a culturally appropriate understanding of clinical decision-making and, more specifically, the deliberation of ethical dilemmas health professionals in such clinical contexts might face. Our study highlighted three major challenges in clinical decision-making in old-age care, namely in congruence between older patients’ religious views and modern medicine, lack of information received by, and informed consent among, older patients, and the strong involvement of families of older patients in clinical decision-making.

Our finding that older patients’ religious values could conflict with treatment recommendations in healthcare facilities is supported by various studies on the effect of patient religious values on treatment adherence in Ethiopia [57], Palestine [58], Jamaica [59], Iran [3], Africa [60] and globally [61, 62]. This finding is also in line with a recent review of experiences of health professionals working with patients who hold rigid religious beliefs in Africa, which “discusses religious or spiritual inclinations that negatively affect healthcare access and adherence, especially to biomedical or western medicine” [60]. We found no specific literature on how older adults’ religious beliefs in Ethiopia influence their decisions to seek or adhere to modern healthcare, except for one cross-sectional study on determinants of health-seeking behaviour among older patients, which only listed participants’ religion as one of the predictors of healthcare-seeking behaviour but did not elaborate further [63]. Nevertheless, several studies [27, 57, 64, 65] reporting on the general adult population’s relatively lower interest in modern medicine than in alternative and traditional healing practices correspond with our health professional participants’ claim that older patients in Ethiopia hold strong religious values that conflict with modern medicine. This is further reflected in theWHO’s 2019 global report on traditional and complementary medicine, which states that the use of traditional medicine in meeting primary healthcare needs in developing countries ranges from 60 to 90% whereas, in developed countries, it is 40% to 50% [66].

Therefore, the major challenge for health professionals working with older patients in such contexts, as also reported by several participants in our study, is balancing the tension between traditional medicine (including cultural and religious healing practices)—which has a long history in Africa [67]—and modern medicine. This tension touches upon broader debates on cultural relativism and universal ethical principles. Cultural relativism, the position that all cultural practices and beliefs must be understood in their context and not judged by external norms, can conflict with universal ethical principles in bioethics, such as beneficence and non-maleficence, which advocate for the best possible health outcomes, regardless of cultural contexts. Navigating this tension requires a nuanced approach that respects local moral underpinnings while striving to provide quality medical care.

An underlying ethical dilemma faced by health professionals interviewed in our study—and which is also reflected in other healthcare ethics literature—is balancing beneficence and autonomy. Health professionals' strong interest in benefiting their (older) patients by promoting adherence to the suggested treatment plan was often contrasted with a lack of interest from patients who may have other priorities. The finding that health professionals attempt to convince older patients is in line with the concept of beneficent persuasion [68, 69] or benign manipulation [70] in clinical practice, which involves employing decision-making psychology by health professionals to initiate patient behaviour that—through the lens of biomedicine—is believed to benefit the long-term health of patients. There are ongoing debates regarding whether the use of persuasion in clinical practice is ethically justifiable. While it is viewed as an unnecessarily paternalistic practice that interferes with voluntariness, there is also an argument that persuasion, especially when done with empathy and respect, helps physicians fulfil their moral duty to enhance patient autonomy and improve patient well-being [68, 71–73]. Based on experiences from Ethiopia, the current study contributes to this discussion by showing that health professionals who, in particular, work with older patients might prioritise benefiting patients over mainly facilitating voluntary decisions among their patients. More broadly, this finding could also reflect the culture of shared or communal decision-making in Ethiopia, which seems to be different from, for example, a Western context where autonomy and individualism are more often the leading principles physicians adhere to.

Our finding regarding balancing beneficence and autonomy is pertinent to global bioethical discussions on paternalism and patient autonomy. In many Western contexts, patient autonomy is a dominant ethical principle in which patients make informed decisions about their own health [74]. However, in non-Western contexts, communal decision-making and paternalism often play a more significant role and there is a tendency to prioritise family and/or community over individual autonomy [12, 15]. For example, evidence shows that in many Asian and African cultures, family members are often involved in decision-making processes, particularly when it comes to serious medical conditions [13, 75]. Such differences across regions highlight the importance of cultural competence in bioethics and recognise that ethical practices must be adaptable to different cultural contexts to ensure both respect for patient autonomy and culturally appropriate care. Moreover, the ongoing globalisation of healthcare further underscores the need for a bioethical framework that is flexible and inclusive of diverse cultural perspectives whereby healthcare providers are equipped to navigate the ethical complexities of a multicultural world. Another finding that gives an additional ethical perspective on clinical decision-making includes paternalistic attitudes such as providing incomplete information and/or white lies. A white lie is defined as “a deceptive interaction to prevent injury or grief or to protect people’s feelings” [76]. The act of paternalistic lying for the benefit of the patient as reported in our study agrees with similar findings of paternalism captured in studies from India [77] and Iran [76], as well as studies from the US [78] and other countries [79, 80]. Although how much information constitutes—or ought to do so—informed consent is a separate discussion necessitating further philosophical deliberation, it is permissible to claim that, especially from a conventional biomedical ethics perspective, lying to a patient is generally ethically problematic as it clashes with the duty to ensure informed consent in healthcare [74]. However, as shown in our study, lying or hiding clinical information from older patients in the Ethiopian healthcare context could be a sign of care by protecting the person from a bitter truth. Although literature explicitly addressing white lies in clinical practice in Ethiopia is non-existent (to our knowledge), one case study among Ethiopian migrants in the US narrates truth-telling in the Ethiopian culture.

It is vital in Ethiopian culture that to break bad news such as grave illness or the death of a family member, an appropriate time, place, and way be chosen. A sudden shock is to be avoided at all cost, especially when dealing with children, the elderly, and sick people because of the harmful effects this news may have on people with fragile emotional states. […] In Ethiopian culture, the patient and healer relationship is paternalistic and protective, and trust is a major component of this relationship. […] Disclosing a serious diagnosis with a poor prognosis conflicts with the hope for healing [81].

On the contrary, another study on breaking bad news to cancer patients in Ethiopia states that contrary to the preferences of family caregivers, patients want to be informed and that balancing this tension “creates an ethical dilemma for staff in terms of how much they involve their patients in clinical decision-making” [82]. This is in line with some older patients in our study who expressed a desire to get detailed information about their treatment and criticised health professionals who did not do that. The ethics of lying to patients remains a debatable issue [83–85] and any further analyses need to be and remain sensitive to the contexts that surround these arguments. This debate ties into the broader bioethical discussion on truth-telling versus therapeutic privilege. In the African context (acknowledging exceptions), the emphasis on truth-telling may be balanced with or even outweighed by considerations of therapeutic privilege—the idea that withholding certain information might be more beneficial to the patient if disclosing it could cause unnecessary harm [14]. The ethical challenge lies in finding a balance that respects patient autonomy while also considering the potential harm that full disclosure might cause, especially in culturally specific contexts where different values and expectations prevail.

Lastly, our findings on family involvement entail interesting ethical perspectives in clinical decision-making. Instead of only family paternalism (when family members withhold from the patient information that they believe could be harmful to the patient), this study suggested that there is family decision-making, where the family’s interests may override patients autonomy rights. Since older adults in Ethiopia depend on their family’s financial and other support to receive healthcare, this reliance is used to justify having families, rather than patients, make healthcare decisions. One interesting implication of this finding is how social health insurance might enhance and enable patient autonomy. Our finding that older patients’ families have strong involvement and influence in decision-making concerning the medical treatment of older patients corroborates what Menon et al. claim: “family involvement in healthcare decision-making for competent patients occurs to varying degrees in many communities around the world” [86]. The strong family pressure on older patients could also explain the paradox in our findings, where some older patient participants expressed a lack of desire to engage with modern medicine, yet they still utilise it. Additionally, our finding suggests that the perception of how health professionals ought to accommodate family involvement may vary depending on the cultural context. In the context of Ethiopia, as our study and as other evidence suggests [87–89], the family often takes a major role in clinical decision-making. However, there were opinions from a few participants in this study against such paternalistic practices. This might imply that Western ideas of patient autonomy are getting more widely adopted in Ethiopia, which requires further investigation.

The strong family involvement identified in this study is also supported by an ethical guideline for physicians in Ethiopia (entitled “Medical Ethics for Doctors in Ethiopia”), which stipulates that physicians should involve the patient’s family from simple communications to decision-making (see Table 2 for relevant articles extracted from the guideline) [90].

**Table 2:** Relevant articles selected from the ethical guideline for medical doctors in Ethiopia

Sections and articles	Page numbers
*I. Doctor-patient and Doctor-community Relationships*	
Article 5: *The doctor shall provide her/his patient, the family and the whole community with the prevention of disease or injury, maintenance of good health and rehabilitative services*	7
Article 7: *The doctor shall make use of every opportunity to teach the patient and her/his family regarding the prevention of disease and the promotion of health*	7
*VI. Certificates, Prescriptions and Signatures*	
Article 34: *The doctor shall formulate his prescriptions with the necessary clarity. The doctor and/or the pharmacist make sure that the patient or his family has well understood the ordered prescription. The doctor will try her/his best to see that the treatment is carried out*	10
*B. Basic Principles for all Medical Research*	
22. *Participation by competent individuals as subjects in medical research must be voluntary. Although it may be appropriate to consult family members or community leaders, no competent individual may be enrolled in a research study unless he or she freely agrees*	26

Medical Ethics for Doctors in Ethiopia (2010), Ethiopian Medical Association [90]

This maybe different from clinical practices in a Western context where, although differences across Western countries also exist [91], family involvement could be less evident as compared with other cultural contexts in, for example, Africa and Asia [92]. Although this study does not engage with a comparative analysis of the different decision-making contexts across cultures, the above finding could inform conventional understandings of effective, culturally competent clinical decision-making.

Although providing a detailed strategy is beyond the aim and scope of this study, a few broad solutions and recommendations may be proposed to address the issues identified. For instance, health professionals could develop more patient-centred approaches that respect the religious beliefs and values of older patients while also ensuring that patients receive adequate medical care. Such approaches may contribute to addressing the challenge of religion interfering with older patients' decisions to seek healthcare services. Given the crucial role of religious leaders in influencing health care utilisation behaviour [93–95], such approaches could involve engaging religious leaders to guide how religious beliefs could coexist with modern medical practices. Moreover, health professionals could receive training on cultural competency and effective communication strategies to better understand and address the cultural contexts of older patients.

Additional measures could aim at balancing the role of family and older patients’ individual interests with regard to clinical decisions affecting the medical care of older patients. Health professionals could work more closely with families to ensure a culturally appropriate balance between the best interests of older patients and family wishes by, among others, facilitating culturally sensitive communication between older patients, families, and health professionals as appropriate. Similarly, the lack of informed consent identified in this study highlights the necessity for implementing policies and procedures that ensure provisions of comprehensive and understandable information to older patients about their treatment, while also respecting and balancing the local cultural context. This could be achieved through culturally sensitive communication strategies, ongoing training for health professionals on ethical decision-making within the local cultural context, and collaborative efforts between stakeholders (health professionals, community stakeholders, older patient representatives, and health policymakers) to develop guidelines that uphold the principles of informed consent while respecting cultural norms in relation to disclosure.

### Limitations

Although this study provides interesting insights into clinical decision-making in old-age care, it is not without limitations. First, as this study was conducted in the capital city (Addis Ababa) and did not include rural settings, it might have missed some unique experiences of clinical decision-making in rural contexts. However, as rural–urban migration to Addis Ababa is rapidly increasing [96, 97], the city likely reflects both urban and rural cultures, and we obtained sufficiently diverse perspectives to meet our exploratory objective. Additionally, as Braun & Clarke pointed out [52], our analysis and presentation in this study could have been shaped by our perspectives, particularly considering the exploratory qualitative methodology used, which lacked stringent theoretical frameworks. Nonetheless, to mitigate this potential bias, all authors engaged in reflective discussions on the emerging themes and rigorously reviewed the manuscript from the initial draft. An additional limitation is that, mainly due to time and resource limitations as a result of the COVID-19 pandemic, we did not interview family caregivers of older patients. This might have added more perspectives on the dynamics of clinical decision-making.

## Conclusion

This study suggests that clinical decision-making can be highly influenced by the given sociocultural context of clinical practice, including religious beliefs among patients. Better and (ethically) robust clinical decision-making in old-age care could contribute to facilitating a smooth decision-making process for both older patients (and their family caregivers) and health professionals and, more broadly, enhance clinical practice with older patients. Many of the findings in this study might also reflect the challenges in clinical decision-making involving other vulnerable patient groups (e.g., patients with disabilities). Thus, health policy efforts in Ethiopia could also aim at improving practices of clinical decision-making involving a wider patient population. Further research could include informal caregivers’ perspectives and compare decision-making challenges among older patients with those among younger patients. In addition, in a context like Ethiopia, where religion and family greatly influence clinical practice with older patients in particular, health professionals will need more ethics and cultural competence training to ensure smooth clinical decision-making.

## Supplementary Information

Below is the link to the electronic supplementary material.

Supplementary file1 (DOCX 15 KB)

## Data Availability

The data used for this study cannot be publicly available due to the risk of compromising individual privacy of study participants. However, the transcripts relevant for this paper can be obtained from the corresponding author upon reasonable request.
