# Supplementary material for: “I Do Not Believe We Should Disclose Everything to an Older Patient”: Challenges and Ethical Concerns in Clinical Decision-Making in Old-Age Care in Ethiopia
Source: Health Care Anal. 2024 Oct 1;32(4):290–311. doi: 10.1007/s10728-024-00494-y (PMC11532312; doi:10.1007/s10728-024-00494-y)
Supplement: Supplementary file 1 — Supplementary file1 (DOCX 15 KB) [file 10728_2024_494_MOESM1_ESM.docx]

**Summary of the interview guides and probes**

**Interview guide for older adults**

1. Introduction and demographic information

- Please tell me about yourself, including your age, work or activities you do, family condition, etc?

1. Deciding about your healthcare

- How would you describe your health condition in general?
- How often do you seek care from healthcare facilities?
- Do you use other means for a cure when you are ill?
- What is the role of religion in this?
- Have you ever preferred religious practices over modern medicine or the other way round? If so, why?
- How much information do you get from health professionals during your treatment?

1. Involving others when deciding about treatment

- Do you have anyone you consult about what to do when you are ill?
- Can you remember any recent example where your family was involved in making decisions for your care and you disagreed? If so, how was the experience?
- Was there a time your family and health professionals decided together for you? If so, how was the experience?

**Interview guide for health professionals**

1. Introduction and demographic information

- Please tell me about yourself, including your age, qualification, work experience, etc.

1. Facilitating decisions for older patients

- When treating older patients, how much information do you give them about their condition, especially compared with younger patients?
- How do you see the involvement of families in making decisions for the care of older patients?
- Do you remember a time that you lied to an older patient? If so, could you explain why and how?

1. Your perception of how older patients make decisions

- What is your opinion concerning older patients’ interest in modern medicine?
- How do older patients’ cultural or religious beliefs impact treatment outcomes?
